# Novel variants of *CYP21A2* in Vietnamese patients with congenital adrenal hyperplasia

**DOI:** 10.1002/mgg3.623

**Published:** 2019-02-27

**Authors:** Dung V. Chi, Thinh H. Tran, Duc H. Nguyen, Long H. Luong, Phuong T. Le, Minh H. Ta, Huong T. T. Ngo, Mai P. Nguyen, Tuan P. Le‐Anh, Dat P. Nguyen, The‐Hung Bui, Van T. Ta, Van K. Tran

**Affiliations:** ^1^ Center for Gene and Protein Research Hanoi Medical University Hanoi Vietnam; ^2^ National Pediatric Hospital Hanoi Vietnam; ^3^ Hanoi Medical University Hospital Hanoi Vietnam; ^4^ Clinical Genetics Unit Center for Molecular Medicine Karolinska Institutet Karolinska University Hospital Stockholm Sweden

**Keywords:** 21OH, congenital adrenal hyperplasia, *CYP21A2*, mutation spectrum, novel variants

## Abstract

**Background:**

Congenital adrenal hyperplasia (CAH) (OMIM #201910) is a complex disease most often caused by pathogenic variant of the *CYP21A2* gene. We have designed an efficient multistep approach to diagnose and classify CAH cases due to *CYP21A2* variant and to study the genotype‐phenotype relationship.

**Methods:**

A large cohort of 212 Vietnamese patients from 204 families was recruited. We utilized Multiplex Ligation‐dependent Probe Amplification to identify large deletion or rearrangement followed by complete gene sequencing of *CYP21A2* to map single‐nucleotide changes and possible novel variants.

**Results:**

Pathogenic variants were identified in 398 out of 408 alleles (97.5%). The variants indexed span across most of the *CYP21A2* gene regions. The most common genotypes were: I2g/I2g (15.35%); Del/Del (14.4%); Del/I2g (10.89%); p.R356W/p.R356W (6.44%); and exon 1–3 del/exon 1–3 del (5.44%). In addition to the previously characterized and documented variants, we also discovered six novel variants which were not previously reported, in silico tools were used to support the pathogenicity of these variants.

**Conclusion:**

The result will contribute in further understanding the genotype‐phenotype relationship of CAH patients and to guide better treatment and management of the affected.

## 1 INTRODUCTION

Congenital adrenal hyperplasia (CAH) is a group of congenital, autosomal recessive diseases defined by cortisol synthesis disorders. The incidence of CAH differs greatly between ethnicity and geographic regions, ranging from 5 to 10 cases per 100,000 live births (Pang & Shook, 1997; Therrell, 2001; van der Kamp & Wit, 2004). The majority of CAH are caused by adrenal steroid 21‐hydroxylase enzyme (P450c21) deficiency (OMIM accession number #201910) (Krone, Dhir, Ivison, & Arlt, 2007; White & Speiser, 2000). The enzyme catalyzes the conversion of 17‐hydroxyprogesterone (17‐OHP) into 11‐deoxycortisol and progesterone into deoxycorticosterone, which are precursors to synthesize cortisol and aldosterone (Turcu & Auchus, 2015). The enzyme dysfunction is mainly tied to its coding gene, *CYP21A2* (OMIM*613815), which lie on locus 6p21.3 among the major histocompatibility complex (MHC). Due to the high variability in the MHC locus, the nearby *CYP21A2* pseudogene (*CYP21A1P*) and gene conversion, it is more challenging to precisely identify the pathogenic variants of *CYP21A2* compare with other monogenic diseases. (White, Tusie‐Luna, New, & Speiser, 1994)

About 75% of CAH cases present severe complications such as salt loss, weight faltering, and life‐threatening hypovolemic shock (Speiser et al., 2010). Clinical classification of CAH divides the disease into three main groups: Classic salt‐wasting, simple virilizing, and the mild nonclassic form (Trapp, Speiser, & Oberfield, 2011). The clinical manifestation and severity of CAH are directly linked with how much 21‐Hydroxylase enzymatic function remains, which mainly result from the pathogenic variants of the *CYP21A2* gene. Without timely diagnosis and intervention, newborns undergo accelerated growth, sexual abnormality and, in cases of severe to complete enzymatic deficiency, potentially fatal salt wasting (SW).

To date, there are over 100 pathogenic variants of *CYP21A2* identified. It has been a general consensus to categorize these variants into several main groups, and the most widely accepted classification is based on the predicted functionality of the 21‐Hydroxylase enzyme (Krone & Arlt, 2009). The NULL group includes genotypes with no enzymatic functionality (i.e., gene deletion, gene conversion, F3061t, Q318X, R356W …); group A contains a pathogenic variant of the intron 2 splice sites (I2G/I2G or I2G/NULL) which result in minimal 21‐Hydroxylase function; group B contains the less severe I172N variant which in in‐vitro experiment have shown a residual 2% enzymatic activity; the remainders group carry either allele with P30L, V281L, and P453S missense variants or variants which result in partial decrease in 21‐Hydroxylase activity.

It is of particular importance to classify and study the genome of the CAH patients, especially with the advance of prenatal diagnosis; this will help prediction of the disease severity and better treatment outcome for the patients. We have designed an efficient multistep approach to diagnose and classify CAH cases due to *CYP21A2* pathogenic variants and to study the genotype‐phenotype relationship. Till date, this is the first cohort of CAH and the most comprehensive investigation of pathogenic variants of *CYP21A2* in Vietnam.

## 2 METHOD

### 2.1 Patient acquisition

A large cohort of 212 patients of Vietnamese descent from 204 families was recruited. Patients with confirmed diagnosis of CAH were part of an ongoing cohort at the National Pediatric Hospital (Hanoi, Vietnam). Complete clinical and genetic profiling was conducted at the time of admission. Genomic DNA was extracted from peripheral blood samples using the QiaAmp DNA blood mini kit following the manufacturer's instructions (Qiagen, Germany).

### 2.2 Clinical parameter

The complete clinical evaluation was performed at admission. Prader Scale was used for measurement of the degree of virilization (Score 0–5). Testosterone, serum 17OHD, and ACTH stimulated 17OHD were recorded at baseline before the patients start hormone therapy, because sometimes healthy preterm neonates may present an elevated level of 17‐OH resulting in false positive. Diagnosis of clinical CAH needs to be made based on both pretreatment biochemical result, and careful follow‐up of the treatment response.

### 2.3 MLPA

Multiplex Ligation‐dependent Probe Amplification (MLPA), a high‐throughput and straightforward technique for quantification of gene copy number was performed using the MLPA Kit P050B2 (MRC‐ Holland) according to the manufacturer's protocol. Products from amplification were analyzed on a 3100‐Avant Genetic Analyzer ABI‐PRISM (ThermoFisher). The kit contains five probes for *CYP21A2* (p.P30L, p.G110fsX21, p.I172N, E6cluster, and p.Q318X), three CYP21A1P‐specific probes, together with two probes for complement components (C3, C4) and is utilized to detect deletions and duplications of one or more exons of *CYP21A2*.

### 2.4 Sequencing *CYP21A2* genes

To identify missense variants in the *CYP21A2* gene, another PCR amplification product (100–150 ng starting DNA) was obtained for each sample. After agarose gel discrimination, the PCR product was purified with Gel Purification Kit followed by sequencing using Big Dye Terminator V3.1 (Applied Biosystems). Results were analyzed by CLC Main Workbench Software with the reference *CYP21A2* on GeneBank (Accession number NM_000500.7). Novel variants were confirmed by conducting search on online databases (i.e., Leiden Open Variant Database [LOVD]) and publications on *CYP21A2* gene. The primers used are provided in Supporting Information. We also performed mutation identification for the parents to determine the cis/trans state of the child variants.

### 2.5 Classification of variant groups

Variants were classified into group NULL, A, B, C based on their projected in vitro enzymatic function and in silico stimulations (Haider et al., 2013; Krone & Arlt, 2009). In addition, group D was for variants with unknown enzymatic consequence and novels, uncharacterized variants.

### 2.6 Data analysis

Data analysis was performed in R (version 3.4.2); statistical significance was defined as *p*‐value <0.05. Statistical parameters are presented as median (min–max) and in the number of cases and percentage (*n*; %). Significance was calculated by a binomial test for two group comparisons (male/female), Wilcoxon rank‐sum test was used for comparing two medians, while Kruskal–Wallis test was used for multiple group comparisons. Polyphen2 was used for in silico analysis of novel variants (Adzhubei et al., 2010).

### 2.7 Editorial policies and ethical considerations

Informed Consent was obtained from the parents of the patient prior to recruitment into the cohort. No identifiable information of the patients was disclosed in any form. This study was reviewed and approved by the ethical committees of Hanoi Medical University (Hanoi Medical University Institutional Review Board, Hanoi, Vietnam; Reference number: IRB00003121).

## 3 RESULT

The current cohort investigated 212 patients from 204 families (eight pairs of siblings) with a confirmed diagnosis of CAH caused by 21‐hydroxylase deficiency. Genotyping and pathogenic variants identification was performed on 204 unrelated patients. With the diagnosis strategy of MLPA to detect deletion or conversion followed by gene sequencing to identify single‐nucleotide changes, we had characterized 202 out of 204 patients and 391 of 404 disease‐causing variants (diagnostic sensitivity 96.8%).

**Figure 1:**
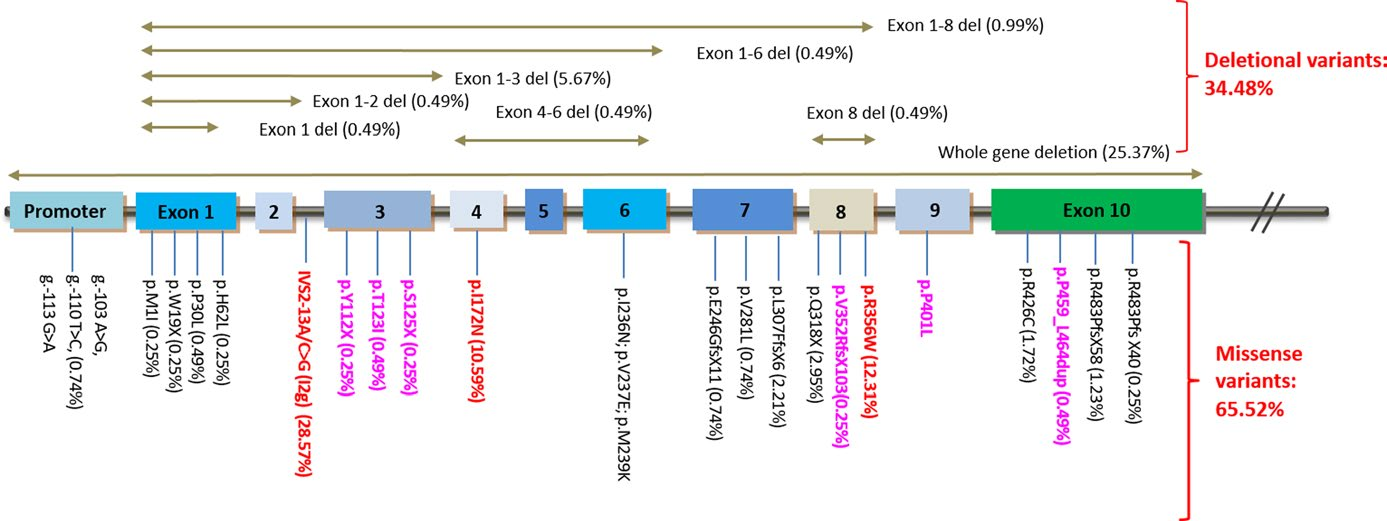
Distribution of the variants on *CYP21A2* in 202 Vietnamese congenital adrenal hyperplasia patients. The number beside each variant indicates its frequency in our cohort (%). Red indicates high frequency variants; Purple indicates novel variants

Figure 1 displays the map of the disease‐causing alleles as well as the occurrence and location of the variants. Using both genetic diagnostic methods (MLPA and Gene sequencing) we have covered the whole span of the *CYP21A2* gene region; the variants indexed spaced across most of the *CYP21A2* gene regions (8/10 exons, promoter and intron 2). The frequency of large deletions was 34.48%, and complete deletion of *CYP21A2* was 25.37%. The hotspots were intron 2 Splice site (I2G) which was detected in 116 alleles (28.57%), exon 8 (15.51%), and exon 4 (I172N) (10.59%). In addition to the characterized and already registered pathogenic variants, we also discovered six novel variants: p.112X; p.T123I; p.S125X; p.V352RfsX103; p.401L; and p.P459_L464dup which were not described in any variant library. The phenotype and biochemical parameters of the cases with novel variants are described in detail in Table 1.

**Table 1:** Genotype and clinical characteristic of six patients with novel variant

Genotype	Clinical manifestation	Gender	Age of diagnosis	Clinical presentations	Polyphen2Prediction/variant classification
p.Y112X/p.I172N	Simple virilizing	Female	14 months	Prader III–IV.17‐OHP 27.7 ng/ml; testosterone 16.3 nmol/L.	N/ALikely pathogenic
I2G/p.T123I	Salt wasting	Female	1 day	Prader III. 17‐OHP 26.8 ng/ml.	Probably damaging score: 0.959Likely pathogenic
p.R356W/p.P401L	Simple virilizing	Female	9 months	Prader II.17‐OHP 28.8 nmol/L; testosterone 1.41 nmol/L.	Probably damaging score: 1.000Likely pathogenic
p.P30L/p.P459_L464dup.	Non‐classic	Female	19 months	Prader I–II.17‐OHP 59 ng/ml.	N/ALikely pathogenic
Del/p.V352Rfs	Salt wasting	Male	26 days	17‐OHP 35.4 ng/ml; testosterone 3.1 nmol/L.	N/ALikely pathogenic
Del/p.S125X	Salt wasting	Male	45 days	17‐OHP 221.4 ng/ml; testosterone 13.9 nmol/L; progesterone 64.9 nmol/L.	N/ALikely pathogenic

Even with strict screening and sequencing process, pathogenic variants in 13/408 alleles (13 patients with one unidentified alleles) were unidentifiable, and these were allocated to group D together along with the novel variants. A total of 55 different genotypes have been identified, 102 patients (50.5%) were homozygotes; 87 patients (43.1%) were heterozygous; 13 patients (6.4%) had only one allele with identifiable variant. The most common genotypes were: I2g/I2g (31/202; 15.35%); Del/Del (29/202; 14.4%); Del/I2g (22/202; 10.89%); p.R356W/p.R356W (13/202; 6.44%) and exon 1–3 del/exon 1–3 del (11/202; 5.44%); 12 patients (5.9%) had variants in a cis‐state (more than one variant on the same allele) (Table 2).

**Table 2:** Genotype of CAH patients

Variant classification	Genotype	Clinical manifestation			*N*	%
		SW	SV	NC		
NULL	Complete deletion (Del/Del)	29			29	14.4
	exon 1 del/exon 1 del	1			1	0.5
	exon 1–2 del/exon 1–2 del	1			1	0.5
	exon 1–3 del/exon 1–3 del	11			11	5.5
	exon 1–8 del/exon 1–8 del	2			2	1.0
	exon 8 del/exon 8 del	1			1	0.5
	exon 1–6 del/exon 4–6 del	2			2	1.0
	Del/exon 1–3 del	1			1	0.5
	p.R356W/p.R356W	12	1		13	6.4
	Del/p.E246GfsX11	1			1	0.5
	Del/p.L307FfsX6	2			2	1.0
	Del/p.Q318X+p.R356W	1			1	0.5
	Del/p.R356W	10			10	5.0
	Del+p.H62L/p.Q318X			1	1	0.5
	Del/p.W19X	1			1	0.5
	p.Q318X+p.R356W/p.R356W	1			1	0.5
	p.Q318X+p.R356W/p.Q318X+p.R356W	1			1	0.5
	p.L307FfsX6/p.L307FfsX6+p.Q318X	1			1	0.5
A/NULL	I2g/Del	22			22	10.9
	I2g/p.Q318X+p.R356W	2			2	1.0
	I2g/p.Q318X	1			1	0.5
	I2g/p.R356W	2			2	1.0
	I2g/p.L307FfsX6	1			1	0.5
A/AA/B	I2g/g.‐113G>A+g.‐110T>C+g.‐103A>G	1			1	0.5
	I2g/I2g	27	2	2	31	15.4
	I2g/p.M1I	1			1	0.5
	I2g/I2g+p.I172N		1		1	0.5
	I2g/g.‐113G>A+g.‐110T>C+g.‐103A>G+I2g+p.P30L	1			1	0.5
	p.I172N/I2g		4		4	2.0
	p.I172N/p.I172N+I2g	1			1	0.5
B/NULL	p.I172N/Del		10		10	5.0
	p.I172N/p.Q318X		1		1	0.5
	p.I172N/p.R356W		2		2	1.0
	p.I172N/p.R426C		2		2	1.0
B/B	p.I172N/p.I172N	1	8		9	4.5
	p.I172N/g.‐113G>A+g.‐110T>C+g.‐103A>G		1		1	0.5
C	p.V281L/p.L307FfsX6			3	3	1.5
D	p.R483PfsX58/p.R483PfsX58	2			2	1.0
	p.R426C/p.R483PfsX40	1			1	0.5
	p.R426C/p.R426C	1			1	0.5
	p.E246GfsX11/p.E246GfsX11	1			1	0.5
	p.I236N+p.V237E+p.M239K/p.L307FfsX6		1		1	0.5
	Del/p.R426C	1			1	0.5
	I2g/?	2	3		5	2.5
	p.I172N/?		2		2	1.0
	p.Q318X/?	2			2	1.0
	p.R426C/?		1		1	0.5
	p.R356W/?	2			2	1.0
	p.R483PfsX58/?		1		1	0.5
Novel	p.P30L/p.P459_L464dup			1	1	0.5
	Del/p.S125X	1			1	0.5
	Del/p.V352RfsX103	1			1	0.5
	p.R356/p.P401L		1		1	0.5
	I2g/p.T123I	1			1	0.5
	p.Y112X/p.I172N		1		1	0.5
Total		153	42	7	202	100

*Note*. CAH: congenital adrenal hyperplasia; SW: salt wasting; SV: simple virilization; NC: non‐classic; ?: unidentified pathogenic variant.

The positive predictive values (PPV) were calculated for the variant groups with known functional consequence. PPV for the NULL group was 97.8% (88/90); PPV for group A was 96.5% (55/57); Group B was 93.75% (30/32); and group C was 100% (4/4); the overall PPV was thus 96.65%. The classification of genotype also had varying impact on the degree of virilization of the female external genitalia, evaluated by the Prader genital stage. Group NULL and A had a higher prevalence of virilization than Group B and C, but there was no definite trend between the pathogenic variants severity with the degree of virilization.

## 4 DISCUSSION

This study has investigated the variants of *CYP21A2* in 204 Vietnamese patients. The CAH patients had been included and monitored in the cohort of CAH patients in the endocrinology department of the Vietnam National Pediatric hospital. As a result, the clinical characteristics are well defined, and the cohort is well suited for variant spectrum study.

In our cohort, we described six novel variants not described previously in any variant databases: p.112X; p.T123I; p.S125X; p.V352RfsX103; p.401L; and p.P459_L464dup. We have searched on variant databases such as LOVD and Human genetic variant database as well as publication on CAH on Pubmed to confirm these variants have not been previously identified. Using in silico prediction tools (MutationTaster, Polyphen2, SIFT, Provean and DUET), we were able to confirm the pathogenicity of these variants. The bioinformatics results are provided in Supporting Information. The phenotypes of these patients were also defined and thus, these variants should be included in the classification for future reference.

The most common pathogenic variant in the cohort were deletion (34.48%), followed by Intron 2 pathogenic variants (I2G) (28.57%), p.R356W (15.51%), and p.I172N (10.59%). The most common deletions were complete deletion (25.37%) followed by exon 1–3 deletion (5.67%), deletions were found on most exon except for exon 9. The percentage of deletion in our cohort was higher than other Asian population (China: 10.8%, Japan: 11.8%) (Asanuma et al., 1999; Lee et al., 1998) and was comparable with some population in Europe (Germany: 27.4%; Krone, Braun, Roscher, Knorr, & Schwarz, 2000), Netherlands: 31.9% (Stikkelbroeck et al., 2003), Sweden: 32.2% (Wedell, Thilén, Ritzén, Stengler, & Luthman, 1994), Denmark: 36.8% (Ohlsson, Müller, Skakkebaek, & Schwartz, 1999), Middle Europe: 30.6% (Dolzan et al., 2005), Czech: 38.6% (Vrzalová et al., 2011), and Australia: 33.5% (Jeske et al., 2009). p.R356W variant in Vietnam was higher than most European populations (0%–9.8%) but was similar with Chinese and Japanese populations (15.4% and 17.6%, respectively) (Asanuma et al., 1999; Lee et al., 1998). The proportion of I2G and p.I172N variants vary significantly between populations. Compared to other Asian populations, I2G and p.I172N variants in our cohort was similar with the Japanese (26.5% and 11.8%) and Australian (34.4% and 11.3%) cohorts but was lower than Chinese population (41.5% and 22.3%) (Asanuma et al., 1999; Jeske et al., 2009; Lee et al., 1998). p.V281L variant is most common in North and South America populations (Marino et al., 2011; New et al., 2013), but in our cohort, the prevalence was low (0.74%). Missense variants span across all *CYP21A2* coding regions except for exon 2 and exon 5, which, to our knowledge are rare, given that only a few case reports mentioned disease‐causing variants on these regions (Dubey et al., 2009; Krone, Riepe, Grötzinger, Partsch, & Sippell, 2005; Usui et al., 2004). The pathogenic variants profile suggested that our cohort does share some resemblance with other Asian populations but with a higher proportion of deletional variants.

While 50.5% of patients were homozygous for the pathogenic variant allele, compound heterozygous genotype was detected in 43.1% of cases. In this study, Sanger sequencing was performed on the whole cohort regardless of the MLPA result, and we discovered 12 patients (5.9%) who had more than one variant on the same allele. Because the clinical presentation of 21‐OH is mostly dependent on the residual enzymatic activity, these patients still mostly comply with the genotype‐phenotype correlation. Notably, one patient's genotype had a total of five variants in which one allele carry an Intron 2 splice site variant (I2g) and the other allele carried a total of four variants (g.‐113G>A; g.‐110T>C; g.‐103A>G; I2g; p.P30L). The patient presented SW phenotype, which correlates well with the predictive pathogenic genotype (I2g/I2g).

We were unable to identify pathogenic variant alleles in 13 patients (6.4%) despite the clinical manifestations and biochemical results suggested 21‐OH deficiency. One explanation for this is that pathogenic variants that occur in the noncoding region of *CYP21A2* can alter the enzymatic function or interact with other variants. This would result in either unidentifiable pathogenic variant or discordance between genotype and phenotype. Furthermore, we did not perform a genetic diagnosis for other genes which can also affect steroid 21‐hydroxylase enzyme synthesis or activity. Other explanation might be that we did not rule out the possibility of the rare cases of 17‐OH CAH and 11‐OH CAH which need further molecular confirmation in a later study.

Using the designated protocol, we were able to achieve up to 96.8% diagnostic sensitivity. The diagnostic sensitivity of our cohort was comparable with the result from Krone et al. (2000) (Sens. 98.7%) and other large cohorts (Jääskeläinen, Levo, Voutilainen, & Partanen, 1997; Wedell et al., 1994). Our results, together with the prior report by New et al. (2013) suggested that MLPA when combined with Sanger sequencing is the preferred methodology, which satisfies both cost‐effectiveness and accuracy. In general, 31/55 (56.4%) genotypes characterized in our cohort had a direct genotype‐phenotype relationship, which was higher than that reported by New et al. with direct correlation found in only 21/45 (46.7%). We also took into account that a variant can have multiple possible clinical outcomes. For instance, Intron 2 variant can sometimes associate with the simple virilization (SV) form even though the main outcome of the variant is SW form. This can be explained by the fact that while I2G (g.665A/C>G) variant can cause incorrect splicing of *CYP21A2* in the upstream 3′ region, some transcripts can correct this aberrant splicing thus the patient retains some enzymatic activity (Higashi, Tanae, Inoue, Hiromasa, & Fujii‐Kuriyama, 1988). Additionally, while p.I172N variant mostly results in SV CAH, a small number of patients, by a so far unexplained mechanism developed SW form.

Our results provided strong evidence for the future usage of the detection protocol in the prenatal screening of CAH in Vietnam. This will provide early detection and prediction of the disease's severity and will help clinician's decision on whether or not to administer prenatal treatment. Because of the unpredictability of some genotypes, careful consideration and consultation need to be made before any decision‐making and treatment administration.

## 5 CONCLUSION

This study is the first in Vietnam to define the pathogenic variant profile of CAH caused by mutation of *CYP21A2*. The results should help in further understanding the genotype‐phenotype relationship of CAH patients and guide better treatment and management of the affected fetuses/children. Future study of CAH in Vietnam should aim at early detection and intervention of the patients to research the relationship between genotypes and treatment response.

## CONFLICT OF INTEREST

The authors declare that they have no competing interests.

## AUTHOR CONTRIBUTION

TVT coordinated the study. DCV, VKT, DHN, LHL, and TVT designed the study. DCV, HTtN, MPN, TPLA, THT provided patient care and collected data. PTL, MHT, DPN performed genetic analysis. LHL performed statistical analysis. DCV, VKT, DHN, LHL, and THB interpreted the results and wrote the manuscript. All authors critically reviewed the report. No writing assistance was provided. DCV, VKT, TVT, LHL had full access to all of the data in the study and take responsibility for the integrity of the data. All authors revised the manuscript critically and approved the final version for publication.
