# Supplementary material for: Novel variants of CYP21A2 in Vietnamese patients with congenital adrenal hyperplasia
Source: Mol Genet Genomic Med. 2019 Feb 27;7(5):e623. doi: 10.1002/mgg3.623 (PMC6503067; doi:10.1002/mgg3.623)
Supplement: Supplementary file 1 [file MGG3-7-e623-s001.docx]

**SUPPLEMENTARY INFORMATION**

Supp. Table: CYP21A2 Primer used.

| Primer | Sequence 5’ – 3’ | Region |
| --- | --- | --- |
| P4 | GGCTTTCCAGAGCAGGGAGTAGTC | Exon 3 |
| P5 | TCTCCGAAGGTGAGGTACCAG | Exon 4 |
| P6 | TCGGTGGGAGGGTACCTGAA | Promoter |
| P9 | AGCTGCATCTCCACGATGTGA | Exon 6 |
| P10 | CTGAGGTACCCGGCTGGCATCGGT | Intron 10 |
| P11 | CCTTCCCACAGCTGCATTCTCATGC | Intron 5 |
| P12 | GTGAGCCTGAGTGCCGGTGAGG | Intron 7 |
| P13 | GCAAAAGGCTCCTTCCCAGCAAC | Intron 7 |
| P14 | CCTGGCTCCAGGAACGATC | Intron 9 |
| P15 | CGTGAAAATGTGGTGGAGGCTGGT | Intron 9 |
